# Major Depressive Disorder Preceding the Onset of Progressive Supranuclear Palsy

**DOI:** 10.4306/pi.2009.6.2.112

**Published:** 2009-06-30

**Authors:** Won-Hyoung Kim, Young-Soo Lee, Seung-Ho Jung, Hye-Jin Choi, Myung-Ji Lee, Min-Hee Kang, Chul-Eung Kim, Jeong-Seop Lee, Jae-Nam Bae

**Affiliations:** Department of Psychiatry, College of Medicine, Inha University, Incheon, Korea.

**Keywords:** Progressive supranuclear palsy, Depression, Psychiatric symptoms, Parkinsonian symptoms

## Abstract

Progressive supranuclear palsy (PSP) is a neurodegenerative disease characterized by vertical supranuclear palsy and parkinsonian symptoms. The neuropsychiatric symptoms of PSP include anhedonia, depressed mood and cognitive impairment. Patients with PSP have an increased risk for developing depressive disorders within the next year. However, it is rare to find that major depressive disorder was the antecedent diagnosis of a patient who was later diagnosed with PSP. We present here a patient who suffered from PSP with repetitive falls, a masked face and dysarthria after developing a major depressive disorder.

## Introduction

Progressive supranuclear palsy (PSP) is a neurodegenerative disease that causes vertical supranuclear palsy and parkinsonian symptoms.^1^,^2^ PSP is the second most common cause of Parkinsonism after Parkinson's disease.^3^ PSP also includes bulbar symptoms,^2^,^4^ anhedonia, depressed mood,^5^ dysexecutive dementia^6^ etc. Depressed mood occurs in approximately 20% of PSP patients.^7^ The patients who are diagnosed with PSP are more likely to develop depression within the next year.^8^ However, there have been few reports of cases with major depression as an antecedent symptom of PSP, which was diagnosed later.

We present here a patient with PSP who was hospitalized due to the presence of recurrent falling, masked face, and dysarthria, symptoms that became evident after development of a major depressive disorder.

## Case

A 51-year-old woman was admitted to the psychiatric department presenting with neuroleptic-induced-Parkinsonism. She had no past history of psychiatric problems. Her depressed mood developed eight months prior to admission. Five months prior to the admission, she began to experience frequent falls. Eventually, her left hand's 5^th^ finger was fractured due to a bad fall, and she was hospitalized at the orthopedic department. Twenty days later, she was transferred to a local psychiatric clinic because her depressed mood was aggravated. She was diagnosed with major depressive disorder without psychotic features.

Although the patient did not report being under stress, her admission notes indicated that her main symptoms included depression, anxious mood, loss of interests, insomnia, and suicidal ideation. Her short-term memory was slightly impaired. No other cognitive examination was performed. The brain MRI obtained at the beginning of hospitalization showed no specific findings.

She was treated with 20 mg of escitalopram for depression and 37.5 mg of quetiapine for insomnia. Dysarthria and masked face then developed; nevertheless, she did not take any other medication because her level of subjective discomfort was not sufficiently severe. She was discharged twenty days after the hospitalization because her mood symptoms showed improvement.

Fifty days before the next admission, her memory impairment became progressively worse and she was disoriented as to time. No depressive mood was observed. The quetiapine was switched to 1.5 mg of risperidone. After switching medication, the masked face and her recurrent falls increased remarkably in intensity.

She visited the emergency medical center because of a right orbital wall fracture that she sustained after falling forward. At that time, she manifested masked face, dysarthria and an unstable posture. The extrapyramidal symptoms were suspected to be caused by her small dose of antipsychotics; therefore, she was admitted to the psychiatric department in order to differentiate her condition from dementia with Lewy Bodies.

**FIGURE 1:**
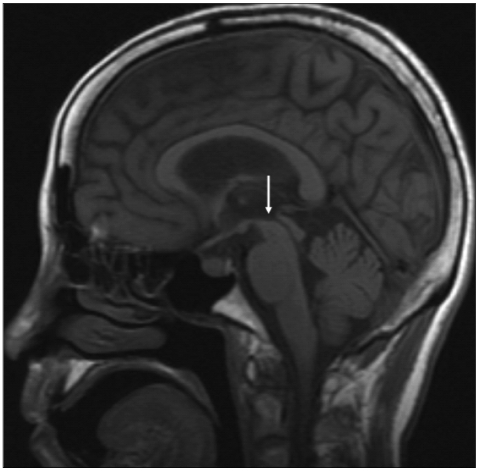
Magnetic resonance imaging. The midsagittal image shows marked atrophy of the midbrain tegmentum and the "penguin silhouette" sign (arrow). The midbrain area was 65 mm^2^ and the pons area was 550 mm^2^. Therefore, the ratio of the midbrain area to the pons area was less than 0.12.

On the mental status examination, she was alert, but disorientation as to time was observed. Her affect was blunted. There were no psychiatric symptoms such as hallucination, delusion, depressed mood or suicidal ideation. The neurological examination showed pathological eye movements with voluntary saccades, vertical gaze palsy, bradykinesia, reduced arm swing, postural instability and a decreased rate of blinking. Ataxia, tremor and dysphagia were not observed. No pathological findings were obtained from the standard laboratory tests, electrocardiography (ECG), chest X-ray and electroencephalogram (EEG). The brain MRI revealed diffuse atrophy of the brain and the so-called penguin silhouette sign (atrophy of the midbrain tegmentum and pons, which resembles a lateral view of a standing penguin with a small head and a large body) on the midsagittal view. The size of the midbrain area was 55 mm^2^, and the ratio of the midbrain tegmental area to the pons area was 0.12 on the midsagittal view (Figure 1).

Upon examination by a neurologist, she was confirmed as suffering from PSP. She scored 17 points on the Minimental status exam, 1 point on the clinical dementia rating, 5 points on the Global Deterioration Scale, 4 points on the Geriatric Depression Scale, 15 points on the Barthel Index (the upper limit was 20) and 35 points on the Instrumental Activities of Living (the upper limit was 45). The neuropsychological examination revealed working memory impairment, disorientation as to time and person, memory impairment and decline of frontal lobe executive function.

Atypical antipsychotics and escitalopram were not used because the Parkinsonism was more severe than the psychiatric and mood symptoms, except for the blunted affect. Six hundred mg of levodopa and 150 mg of decarboxylase inhibitor were used to treat the Parkinsonism. Although the Parkinsonism was not improved, she was transferred to the chronic geriatric hospital because of her poor economic status.

Two months after the discharge, we performed a follow-up exam with permission from her family. Three hundred mg of levodopa, 75 mg of decarboxylase inhibitor and 10 mg of selegiline were used to treat the Parkinsonism. Masked face and hypophonia were observed. She was not depressed. The neurological examination showed vertical gaze palsy, voluntary saccades, a decreased blinking rate, stiff gait and bradykinesia. She had stopped falling down because of her improved postural stability. The mini-mental status exam score was 21 points. Disorientation as to time, concentration impairment and dyscalculia had persisted, while orientation to place and her memory recall were improved.

## Discussion

The National Institute of Neurological Disorders and the Society for Progressive Supranuclear Palsy (NINDS-SPSP)^9^ diagnostic criteria for "probable" PSP require that falls have occurred within the first year of symptom onset and that there is a vertical supranuclear eye movement disorder.

Psychiatric symptoms for patients with PSP are common, yet the psychopathology of PSP is unclear because the psychiatric symptoms of PSP patients do not have specific features, and the patients with PSP are mainly treated by neurologists.

The likelihood of developing depression increased within the next year after diagnosis of PSP.^8^ In this case, the patient's depression developed three months before the onset of PSP with the symptom of recurrent falls. We could not define whether depression was a first symptom of PSP or if the depression was concurrent with PSP. Quante et al.^10^ reported on one case for which depression preceded the onset of PSP. Because depression developed in the absence of a specific stressor, considering the report of Quante, we could assume that the depression of our patient was the first sign of PSP.

Seventy-seven percent of PSP patients display apathy without depression.^11^ Apathy is often misdiagnosed as depression, which explains why antidepressants are ineffective. In this case, the depressive symptoms were improved by antidepressants, but the apathy persisted.

In this case, making the proper differential diagnosis with the dementia of the Lewy Bodies was needed because of the patient's Parkinsonism, repeated falls and severe neuroleptic sensitivity.^11^,^12^ Yet the vertical gaze palsy and voluntary saccades were obvious. Further, the brain MRI demonstrated the penguin silhouette sign, which was defined as marked atrophy of the midbrain tegmentum, 55 mm^2^ for the midbrain area, and a 0.12 ratio of the midbrain tegmentum area to the pons area on the midsagittal MRI. The midbrain area of <70 mm^2^ (sensitivity=100%, specificity=91.3% for PSP) and the ratio of the midbrain tegmentum area to the pons area of <0.15 (sensitivity=100%, specificity=100% for PSP) strongly suggests a diagnosis of PSP.^11^ Consequently, her symptoms of dementia were from the PSP.

The reduced midbrain area and the ratio of the midbrain area to the area of the pons correlate with the duration of PSP. However, it is not clear when the findings of the brain MRI began. The study of Oba et al.^13^ showed a midbrain area less than 70 mm^2^ for a subject who was diagnosed with PSP 4 months previously. Hence, the specific findings of PSP could appear in a patient for whom the onset was less than 1 year previously. There was a limitation in our case in that the brain MRI was not reviewed when the depression first developed. If the brain MRI could have been reviewed, then the onset of PSP would have become clearer.

The treatment for PSP is to identify the target symptoms so that conservative management can be carried out. Levodopa can be used to decrease the Parkinsonism, and forty to fifty percent of patients treated with Levodopa show modest improvement.^11^ Antidepressants help to improve mood^2^ and methlypenidate can reduce the apathy.^14^ In this case, escitalopram improved the depressed mood, and combined levodopa and carbidopa therapy reduced the features of Parkinson disease. The cognitive functions were improved without treatment, which might have been transient, and so follow-up study was needed. The patient's depression could recur because the antidepressant meds were stopped.

We present here a patient who displayed depression as a first sign of PSP. Awareness of the possibly preceding psychiatric aspects like depression as the first symptom of developing PSP may help in the future to improved treatment. Further investigations should focus on the causality and the mechanism that underline the association between PSP and major depressive disorder.
